# Value innovation: an important aspect of global surgical care

**DOI:** 10.1186/1744-8603-10-1

**Published:** 2014-01-06

**Authors:** Michael Cotton, Jaymie Ang Henry, Lauren Hasek

**Affiliations:** 1 International Collaboration for Essential Surgery (ICES), London, UK; 2 ASGBI Offices, Royal College of Surgeons of England, 35-43 Lincoln's Inn Fields, London WC2A 3PE, UK; 3 University of California Berkeley School of Public Health, Berkeley, USA; 4 University of Pittsburgh, Pittsburgh, USA

**Keywords:** Global surgery, Essential surgery, Value innovation, Surgical technology, External fixator, Laparostomy, Ventriculo-peritoneal shunt, Mesh hernia repair, Intra-ocular lens, Flutter valve

## Abstract

**Introduction:**

Limited resources in low- and middle-income countries (LMICs) drive tremendous innovation in medicine, as well as in other fields. It is not often recognized that several important surgical tools and methods, widely used in high-income countries, have their origins in LMICs. Surgical care around the world stands much to gain from these innovations. In this paper, we provide a short review of some of these succesful innovations and their origins that have had an important impact in healthcare delivery worldwide.

**Review:**

Examples of LMIC innovations that have been adapted in high-income countries include the Bogotá bag for temporary abdominal wound closure, the orthopaedic external fixator for complex fractures, a hydrocephalus fluid valve for normal pressure hydrocephalus, and intra-ocular lens and manual small incision cataract surgery. LMIC innovations that have had tremendous potential global impact include mosquito net mesh for inguinal hernia repair, and a flutter valve for intercostal drainage of pneumothorax.

**Conclusion:**

Surgical innovations from LMICs have been shown to have comparable outcomes at a fraction of the cost of tools used in high-income countries. These innovations have the potential to revolutionize global surgical care. Advocates should actively seek out these innovations, campaign for the financial gains from these innovations to benefit their originators and their countries, and find ways to develop and distribute them locally as well as globally.

## Background

Limited human and physical resources in low- and middle-income countries drive tremendous innovation. Reverse innovation, first popularized by Vijay Govindarajan and Chris Trimble in the business world [1], describes innovations that were originally developed and used in LMICs but later ‘trickled up’ to richer countries. We prefer to use the term ‘value’ innovation, since ‘reverse’ or ‘bottom up’ innovation may belie the importance and quality of these types of innovations. ‘Reverse’ also implies that the natural flow of innovation should be from high to low income settings and suggests that it is unnatural for innovation to emerge from resource- constrained settings. An alternative term may be “cost-efficient innovation”. The essential truth is that these innovations are as good, if not better, in dealing with the task at hand, but come at a fraction of the cost, hence providing tremendous value.

The impetus for the development of low-cost, quality innovation in LMICs is an acute need in the local setting, *not* the potential for export back to rich countries. Yet, the need for low-cost high-quality innovation in medicine knows no borders. Bismarck implied that the turmoil, scarce resources and high demands of war produce innovations of lasting value when he said, “*Krieg ist der Vater alle Dinge*” (War is the Father of All Things). Scarce human and physical resources in many LMICs in the face of dire need have also driven tremendous innovation without sacrificing efficacy. Mounting medical costs around the world urgently demand that we search for value innovation everywhere.

Surgical tools, in particular, have benefited from the creative intelligence of physicians practicing with limited resources around the world. There have been several surgical innovations, now standard therapy worldwide, whose origins are from LMICs. In the following paper we detail some of these surgical innovations, their origins, applicability to high-income settings, and their realized or potential impact in improving healthcare delivery around the world.

### Surgical value innovations

#### Bogotá bag

In 1984, Dr. Oswaldo Borraez was a second year surgical resident at the Hospital San Juan de Dios in the poorest areas of Bogotá, Colombia. He was assisting at a laparotomy for a patient who had a serious abdominal infection, precluding closure. Spotting a three-liter polyethylene urine bag, he suggested its use to close the abdomen as an interim measure. The bag was attached temporarily to the edges of the patient’s abdominal wound and functioned as a patch over the large laparostomy until, at a later time, the abdomen could be formally closed. Previous utlization of other substances for this purpose, such as sterile towels, was known to increase infection rates. The experiment worked; no infection ensued, the transparent bag allowed for direct visual monitoring of the abdominal contents and the bag itself proved to be strong and resilient, yet flexible [2]. An American trauma surgeon, after visiting Colombia and learning of the technique, wrote about the innovation and after numerous studies validating its efficacy [3,4], it soon became popular owing to its obvious simplicity [5,6]. The subsequent addition of low-grade suction improved its efficacy [7]. This method is now a recommended technique for the management of an open abdomen [8]. In 1999, it was found that 25% of surgeons used the Bogotá bag as their preferred method of temporary abdominal closure, being the most common technique for leaving an abdomen open at the time [9]. A 3-liter intravenous infusion “Bogotá” bag costs well under US$5, while polypropylene vacuum bags and synthetic, absorbable meshes depending on their size can cost from US$153 to US$1600 [10]. This simple, highly efficient and cost-effective method of managing complex abdominal injuries, wounds and infections has helped thousands of trauma patients globally. It has been the starting-point for the development of newer, more sophisticated, devices.

### Orthopaedic external fixator

Dr Gavril Abramovich Ilizarov was an orthopedic surgeon who trained in Crimea, Ukraine, but was sent to practice in Kurgan Oblast in far Siberia, where conditions were harsh and facilities resembled those of LMICs. There, in the 1950’s, he developed a piece of “meccano” which resembled a tubular structure with pins attached [11]. Following small skin incisions, these pins are inserted into intact bone on both sides of a fracture, allowing the external structure to hold and stabilize bone alignment. Known now as an external fixator, it has become one of the mainstays of operative fracture treatment [12]. This technique permits minimal interference to blood supply of the bone, direct observation of the wound, ability to monitor and debride an open fracture as necessary without losing stabilization, and is simply and rapidly applied non-traumatically [13-15]. External fixation is recommended in cases of open or closed articular fracture with severe soft-tissue compromise, when the external fixator can be applied in a joint-bridging fashion [16]. It is ideal where there is a higher risk of infection and has long been a useful device for managing such injuries and remains the gold standard. In closed fractures, external fixation is indicated for temporary bridging in severe polytrauma [17,18] and severe closed soft-tissue contusions or degloving injury.

It was only in the late 1980s that Ilizarov traveled to the US to speak of his invention, which was largely unknown till then. Since then, the use of the external fixator has found its place in the armamentarium of every orthopaedic surgeon. External fixators cost between US$1,000 and US$4,000 [19]. Reuse of an external fixator can reduce the mean hospital cost by 25% [20] to 34% [21]. The versatility of this device has spawned local production: an Indian external fixator costs only *c*. US$12 [22]. Sadly, Ilizarov himself gained little financially from his innovation [23].

### Hydrocephalus drainage uni-directional fluid valve

Dr Salomón Hakim was a Colombian neurosurgeon who studied normal-pressure hydrocephalus (NPH), a condition that was previously thought not to exist. He first identified the syndrome in 1957 and, in his workshop at his home in Bogotá, Colombia, produced a functioning uni-directional valve to drain the accumulated cerebrospinal fluid (CSF) that occurs in affected patients. The valve unit has entrance and exit valves respectively, utilizing a corrugated or convoluted spring element for biasing a trapped ball against a fluid inlet seat of the valve. He finally published his work in 1964 and demonstrated the successful treatment of a patient in the USA using his device [24]. Subsequent modern ventriculo-peritoneal shunts for the drainage of CSF, including programmable and self-adjusting types, have been based on the “standard Hakim mechanism” [25]. The modern Codman-Hakim Micro Precision Valve shunt system costs US$650. However, the Indian company Surgiwear produces the Chhabra Micro Precision shunt system for only US$35. A study of hydrocephalic children in Uganda found these two ventriculoperitoneal shunt systems equally effective [26].

### Intra-ocular lens & manual small incision cataract surgery

Growing up in a remote mountain village in the Northeast of Nepal where the nearest school was a 12 days’ walk away, Sandik Ruit knew deprivation and poverty. Determined to qualify as an eye surgeon, he remained motivated to help improve the eyesight of over 50 million people in LMICs suffering with moderate to severe visual loss from cataract. The standard procedure, current at the time, of cataract extraction and provision of +10 lenses gave poor results, and the technique of intra-ocular lens (IOL) implantation, used in HICs, which involved phaco-emulsification by a special instrument and the implantation of an artificial lens, was very expensive. Dr Ruit realized that an alternative was needed. In 1986, he and an Australasian ophthalmologist, Dr Fred Hollows, made highly effective cataract surgery affordable to the poor. They developed ‘Small Incision Cataract Surgery’ (SICS), a suture-free technique that avoids phaco-emulsification and removes a cataract through two very small incisions. Furthermore, in 1995, Dr.Ruit also began to produce an international standard IOL locally with drastically lower production costs that reduced its purchasing price from US$100 to US$3.50c [27]. Studies have found SICS to be “significantly faster, less expensive, and less technology dependent than phaco-emulsification” [28]. While both achieve excellent outcomes with minimal complications, SICS can cope better with higher patient volumes and outpatient care [29].

### Mosquito net mesh for inguinal hernia repair

The Lichtenstein tension-free method of inguinal hernia repair has been shown in studies in many countries to be preferable in providing better outcomes in comparison to other previously popular techniques, such as the Bassini method [30,31]. Yet, while it may be preferable, the Lichtenstein method requires the insertion of a small piece of non-absorbable mesh to bolster the hernia defect. At over US$108 per patient, commercially produced mesh is unaffordable for the majority of hernia patients living in LMICs [32]. Fine nylon mesh, originally used in the manufacture of fancy shoes in Hong Kong, was actually already proposed as an implant by Stock in 1954 [33]. In many LMICs, the manufacture, sale and use of protective mosquito netting is ubiquitous. Tongaonkar and colleagues, rural surgeons in India, validated its use for hernia repair in an excellent 5-year follow-up study [34]. Their innovation was studied in a randomized controlled trial in Burkina Faso [32], which showed no significant difference in the clinical short-term outcome of both types of mesh, with the mosquito net costing 0.004% of the commercially-available mesh. When mosquito net mesh is used, tension-free inguinal hernia repair is approximately one third the cost compared to the use of the conventional alternative [35]. This has been confirmed by a meta-analysis, which also demonstrated no increase in septic complications [36]. Subsequent studies have suggested that mosquito nets might actually be better in terms of bursting strength and anisotropy than commercial products in terms of retraction of the mesh [37].

### Flutter valve for intercostal drainage

The flutter valve is a one-way valve that can be used to allow air or fluid to drain out of the thorax in cases of hemo- or pneumo-thorax [38], without air being drawn back into the pleural cavity on normal breathing. The usefulness of this was realized through experience in Karachi, Pakistan and tested in London [39]. An early report had already realized its usefulness in the war scenario, allowing a patient to ambulate freely [40]. This, costing less than US$5 [41], prevents both pneumostasis and the hazards of breakable bottles, as well as the expense of complex drainage systems, at least ten times as expensive as a sterilized flutter valve. Additionally, after lung reduction surgery, the flutter valve is used to treat prolonged air leaks without the potentially damaging effect of suction on a severely emphysematous lung [42], and is more effective in dealing with postoperative air leaks when there is no persistent pleural space [43]. Also in cases of *pneumocystis carinii*-related pneumothorax, it is very useful [44]. The cost-effectiveness and practicality of the flutter valve is largely the result of needing a less invasive procedure that permits outpatient treatment and shortened hospital stays [45,46]. Campisi and Voitk found outpatient care for spontaneous pneumothorax alone saved one Canadian hospital more than US$16,000 in the 1993–1994 fiscal year [47].

## Conclusions

Meeting the medical needs of large numbers of people, many with little or no resources, is a moral obligation. To satisfy this demand, simplicity of care is an obvious advantage. Medical technology has enabled unimagined advances, but often at great cost financially. In the circumstances where such technology is unavailable or unaffordable, innovations proliferate. Consequently, technology is produced that is not only simpler (and therefore usually less expensive), but that is more appropriate to the local environment. The realization is dawning that the criterion of simplicity may actually be advantageous in high-income countries (HICs), not just because the maintenance of expensive technology is in itself complex, and financially draining, but because if a simple device is as good as a complicated one, then the financial savings made are hugely relevant. Furthermore, if a simple device is more robust and easier to use, and is more effective clinically, then again its advantage is evident.

As we have seen in the surgical world, innovations developed for and implemented in LMICs are usually far less costly than those techniques and technologies that are used in high-income settings. Though innovation can occur anywhere, there exists much original thinking and spontaneous inventiveness amongst many people grappling in sub-optimal environments. The Global Surgical community of surgeons, anesthesiologists, obstetricians, nurses, and administrators alike should actively search for and validate the efficacy of innovations introduced in LMICs, push for the acceptance of these methods and campaign with ferocity for the financial gains of innovations from LMICs to benefit those who have invented and pioneered them. This means, in particular, that healthcare providers from HICs traveling to LMICs to provide medical care should be open and responsive to the technology present before assuming the superiority of developed world technologies in every setting.

Interested institutions in HICs may provide start-up finance for potentially useful developments and contribute to research into their efficacy prior to establishing clinical trials to prove their worth. For this to happen, a forum for sharing ideas needs to be created, but unless the benefits of such innovations developed are to remain in HICs, this forum must arise within LMICs.

Important, innovative methods and devices have the potential to improve outcomes in both HICs and LMICs, and demonstrate significant cost-savings in the setting of ever-increasing costs of healthcare. In resource-limited settings these cost savings translate immediately to lives saved, because cost is a major barrier to treatment. In HICs, however, the impact of ever-increasing costs of medical care must ultimately drive the search for simpler, but equally effective, technologies.
